# How well can you tell? Success of human categorisation of horse behavioural responses depicted in media

**DOI:** 10.1017/awf.2024.55

**Published:** 2024-11-11

**Authors:** Katrina Merkies, Katelyn Trudel

**Affiliations:** Department of Animal Biosciences and Campbell Centre for the Study of Animal Welfare, University of Guelph Guelph, ON N1G 2W1, Canada

**Keywords:** affective state, animal welfare, human-horse interactions, interoception, overt behaviours, subtle behaviours

## Abstract

Horses employ a range of subtle to overt behaviours to communicate their current affective state. Humans who are more cognisant of their own bodily sensations may be more attuned to recognising affective states in horses (*Equus caballus*) thereby promoting positive human-horse interactions. This study investigated human ability to categorise human-horse interactions depicted in media relative to equine behaviour experts and compared participant scores to their level of interoception. Using an online survey, participants (n = 534) categorised 31 photographs and videos as (overt) positive, likely (subtle) positive, neutral, likely (subtle) negative or (overt) negative human-horse interactions from the horse’s point of view and completed the Multidimensional Assessment of Interoceptive Awareness questionnaire (MAIA-2) to assess their level of interoception. Demographic information was also collected (age, gender, education, level of experience with horses, location). Participants differed from expert categorisations of horse affective states across all categories, exactly matching experts only 52.5% of the time and approximately matching experts for positive and negative valence 78.5% of the time. The MAIA-2 did not predict participant ability to accurately categorise human-horse interactions. Women outperformed men in categorising overt positive, overt negative and subtle negative human-horse interactions. Increased levels of education and greater experience with horses were associated with improved categorisation of certain human-horse interactions. More training or awareness is needed to recognise behavioural indicators of horse affect to guide appropriate human-horse activities that impact horse welfare.

## Introduction

The modern human-horse relationship spans a large spectrum of interactions which can be characterised by the context and duration of the contact (Goodwin 1999; Hausberger *et al.* 2008). Some human-horse interactions, such as veterinary care or farriery, are brief and infrequent — but still contribute to a horse’s perception of human interactions (Hausberger *et al.* 2008). Conversely, more long-term human-horse interactions, like ownership, allow the development and maintenance of the human-horse bond (Hausberger *et al.* 2008; Merkies *et al.* 2024). Horses (*Equus caballus*) involved in riding lesson programmes or in equine-assisted services may experience a degree of consistency in their connection with students or clients across multiple sessions or programmes but also a regular turnover in clients creating a mixture of both long- and short-term human-horse interactions (Hausberger *et al.* 2008; Arrazola & Merkies 2020; Ekholm Fry 2021). Due to this repeated turnover of human participants, it is imperative to ensure that human-horse interactions are on the whole positive, as a horse that experiences frequent negative interactions with people may become fearful or resistant, making them more difficult to handle, risking both human and horse safety (Waiblinger *et al.* 2006; Luke *et al.* 2022).

From the horse’s point of view, interactions with humans involve most of their senses: visual, tactile, olfactory and auditory (Rørvang *et al.* 2020; Merkies & Franzin 2021). Individual horses may perceive each interaction as positive, neutral or negative, however we cannot rely upon self-reporting to confirm these subjective emotions as we do in humans; instead we must rely upon the horse’s outward expressions of behaviour as a proxy for their true emotional valence (Russell 2003). Horses are highly expressive animals, and their affective state can be inferred by analysing both observable and measurable qualities, such as their physical behaviours and vocalisations, or physiological markers such as heart rate (de Santis *et al.* 2017; Merkies & Franzin 2021). Physical messaging can range from obvious signals like biting or kicking to subtle signals like changing ear positioning or tightening of the lips (McDonnell & Haviland 1995; Pierard *et al.* 2019). Correctly deciphering these behaviours is central to inferring the valence of the horse’s affective state (positive, neutral or negative) and thereby encouraging positive interactions for both the horse and the human while protecting human safety and improving the overall welfare of the horse (Ladewig 2019).

Affiliative behaviours are indicative of long-term positive affective states in farm animals (Hausberger & Muller 2002). They promote welfare through building the human-horse bond and reducing the incidence of aggressive behaviours (Boissy *et al.* 2007). Animals that exhibit affiliative behaviours may be labeled as ‘relaxed’, ‘friendly’, ‘affectionate’, ‘playful’ or ‘social’ by their caretakers (Boissy *et al.* 2007). Affiliative behaviours from horses directed toward humans can include allogrooming, approaching, olfactory investigation, following human direction, nudging or licking (Pierard *et al.* 2019; Arrazola & Merkies 2020). Within these physical indicators of positive affective states, there are subtleties that may not be immediately noticeable to someone unfamiliar with horse behaviour, like slight turning towards a human or loosening of the lower lip (Arrazola & Merkies 2020; de Oliveira *et al.* 2021).

Agonistic behaviours are characterised by the use of force or aggression to terminate an interaction and when observed it can then be assumed that the current interaction was negative or unpleasant for the instigating horse (McDonnell & Haviland 1995; Briefer Freymond *et al.* 2013; Ekholm Fry 2021). Typically, horses only exhibit agonistic behaviours to the minimum degree required to terminate an unpleasant interaction prior to returning to a normal state, but behaviours will escalate as required until the negative interaction ceases (Briefer Freymond *et al.* 2013; Burla *et al.* 2016). Agonistic behaviours can include both the action itself or the threat of the same action (McDonnell & Haviland 1995; McGreevy 2012). For example, a bite is a common agonistic behaviour across a variety of species (e.g. dogs, cats, horses), but the threat of a bite also represents an agonistic behaviour even if no contact is made (McDonnell & Haviland 1995; McGreevy 2012). Other agonistic behaviours include: (threat of) kicking, (threat of) striking, alert posture and approach, pinned ears, head threat (head lowered with neck extended and ears pinned) or tightened facial musculature (McDonnell & Haviland 1995; McGreevy 2012; Pierard *et al.* 2019). More passive avoidance strategies to cease an interaction may be indicated by subtle behaviours like turning away to increase distance between the horse and the stressor, even though the horse may not be fully removed from the situation (McDonnell & Haviland 1995; McGreevy 2012; Pierard *et al.* 2019; Ekholm Fry 2021). Both agonistic and avoidant behavioural responses exhibited by horses are assumed to be indicative of a negative affective state and embody the physical display of the fight or flight response (Ekholm Fry 2021).

The physical expression of positive or negative affective states are subjective to each individual horse’s temperament but dampened by training which acts to suppress unwanted behaviours (Seaman *et al.* 2002; McGreevy *et al.* 2009). Within these individual differences horses may be categorised as either ‘active’ or ‘passive’ copers (Wechsler 1995; Seaman *et al.* 2002). The major difference between coping styles is that passive copers do not present major outward signs of aversion to stimuli, with agonistic or avoidant behaviours being muted (Wechsler 1995; Seaman *et al.* 2002). In this passive and often immobile state, animals are attempting to go undetected while waiting for the aversive stimuli to pass (Wechsler 1995). This passive reaction may be mistaken for a calm state, as no obvious negative behaviours are detected, but may not be a true representation of the horse’s affective state (Wechsler 1995; Squibb *et al.* 2018). On the other hand, active copers may overtly display their response to a stressor (Budzyńska 2014) which can alert the human to change their approach to a situation.

It is advantageous for humans to be aware of various behaviours their horse displays, nonetheless they are often oblivious to them, especially the more subtle behaviours (Bell *et al.* 2019). When assessing an animal’s emotional state, individuals may bias their evaluation through their personal, subjective lens and imbue their observations with anthropomorphism (Bradshaw & Casey 2007; Thompson & Clarkson 2019). The complexity and subjectivity when assessing an animal’s affective state has been flagged as an issue, and even when using accepted behavioural markers, many studies and welfare assessments have struggled with inter-observer reliability (Green & Mellor 2011; Yeates 2011).

Empathy is the ability to relate to another being’s emotions which implies that one is aware of their own bodily sensations to be able to resonate with another being (Marson *et al.* 2024). Humans more readily empathise with other beings viewed as similar or that they can relate to, and anthropomorphism may act as a catalyst to endow other beings with human qualities thereby making them more like us (Vanutelli & Balconi 2015). As Thompson and Clarkson (2019) argue, the application of anthropomorphism may actually strengthen interspecies relationships as we can see that horses are like us in some ways, although unlike us in others. Empathy involves processing and recognising one’s own internal states, making interoception an important component of empathy (Ernst *et al.* 2013). Interoception is defined as the ability in which one can sense and interpret what is going on within their own body, which can include either conscious or subconscious processes (Mehling *et al.* 2018). Neural pathways involved in affective states are linked to neural pathways involved in physiological responses to the point that interoceptive awareness is believed to drive emotional behaviours meant to fulfill social needs (Craig 2016). Those possessing a higher level of bodily awareness are likely to be more empathetic (Li *et al.* 2024). Specifically, those with a higher interoceptive sensibility were better able to recognise facial expressions of emotions in other people (Hübner *et al.* 2021).

This study investigated whether humans with a higher self-awareness of their own bodily state are better able to recognise a horse’s affective state. To the authors’ knowledge, the influence of interoception on the evaluation of affective states of horses has not been researched. Using a survey methodology whereby participants viewed a number of media depicting horses interacting with humans in various scenarios, their ability to recognise behavioural cues indicating positive, neutral and negative affective states in horses was compared to expert evaluations of each scenario. The percent agreement of participant responses was then related to their scores on the Multidimensional Assessment of Interoceptive Awareness (Version 2) (MAIA-2). The MAIA-2 is one of the most popular validated psychometric scales that serves as a self-reported measure of the participant’s interoception (Vig *et al.* 2022). The 37 MAIA-2 items represent eight subcategories, termed factors, of interoception: noticing, not-distracting, not-worrying, attention regulation, emotional awareness, self-regulation, body listening and trust (Mehling *et al.* 2018). It was hypothesised that those scoring higher in the MAIA-2 would better categorise interactions between humans and horses because they are more aware of their own emotional responses and affective states.

## Materials and methods

### Ethical approval

This research protocol was approved by the institutional Research Ethics Board for the use of humans in research (REB21-12-026).

### Survey development: Media selection and categorisation

All media (photographs and video) were collected from researchers’ personal files. Media depicted positive, negative and neutral horse behavioural responses to humans in a variety of human-horse interactions. Media were selected to portray both overt and subtle responses from the horse. The overt category contained obvious affiliative, agonistic or avoidant behaviours, such as a horse trying to bite a human. Conversely, the subtle category contained less noticeable physical responses from the horse such as a horse backing away while the human is trying to place a halter on their head. To account for potential dampened expression of positive or negative behavioural responses due to the horse’s training, and at the suggestion of the experts, instances of passive coping were grouped as neutral responses to human-horse interactions.

An initial media bank (n = 77 pieces) was distributed to seven external equine behaviour experts for validation to confirm the media categorisations. External experts comprised equine behaviour researchers who had an average of 50 peer-reviewed articles focused on horse behaviour. For media categorisations to be considered validated, at least 85% of the experts (i.e. six of the seven external validators) must have agreed with the categorisation. The final survey contained 31 pieces of media (Table 1) which included 22 videos and nine images.

**Table 1.** Brief descriptions of selected media as agreed upon by equine behaviour experts (n = 7) and included in the final survey to assess how well humans can recognise horse behavioural responses to human interactions. Categories of affective state were divided into positive, negative and neutral, with overt and subtle scenarios for positive and negative situations. Media explanations describe what the horse is doing in the scenario in response to human interactions

Categorisation	Description	Media type	Video length (s)
Overt positive	Horse following target for food reward	Video	8.0
Overt positive	Foal sniffing human crouched down	Video	7.5
Overt positive	Horse being embraced by human	Image	—
Overt positive	Horse lowering head towards human	Image	—
Overt positive	Horse drinking water from hose held by human	Video	8.0
Overt positive	Allogrooming during human petting	Video	9.0
Overt positive	Horse following target for food reward	Video	8.0
Subtle positive	Horse reaching toward humans over fence	Image	—
Subtle positive	Olfactory investigation of human	Image	—
Subtle positive	Horse reaching neck over stall door towards human	Image	—
Subtle positive	Horse reaching neck over stall door towards human	Image	—
Subtle positive	Horse angling head and neck towards human	Image	—
Subtle positive	Horse resting head in human’s arms	Image	—
Neutral	Horse having ear bonnet put on	Video	5.0
Neutral	Horse standing for human to mount	Video	8.0
Neutral	Horse standing in crossties	Video	8.0
Neutral	Horse having hind foot picked out	Video	5.0
Neutral	Horse being bridled	Video	9.0
Neutral	Horse being cross-tied	Video	11.0
Neutral	Horse being bridled	Video	8.0
Subtle negative	Horse being ridden through gate	Video	6.0
Subtle negative	Horse being ridden through water	Video	4.0
Subtle negative	Horse being caught in field	Video	7.0
Overt negative	Horse being asked to cross tarp on ground	Video	10.0
Overt negative	Horse being loaded onto trailer	Video	10.0
Overt negative	Horse being brushed	Video	10.0
Overt negative	Horse having bit size measured	Video	10.0
Overt negative	Horse being petted over stall door	Video	7.0
Overt negative	Horse avoiding being caught in paddock	Video	20.0
Overt negative	Horse raising head high away from human	Image	—
Overt negative	Horse being sprayed with fly spray	Video	9.0

The videos ranged from 4–20 s in length, averaging 8.5 s each, with a total running time of 187.5 s (approximately 3 min). All human faces or identifying features were blurred to respect the privacy of those depicted in the media. In some instances, other horses that were not interacting with the humans in frame were also blurred to avoid any confusion for participants regarding which horse they should be assessing. To focus on the human ability to read solely the physical responses of horses, only visual cues were explored, and all audio was removed from videos to avoid influence from horse vocalisations (e.g. whinny, nicker, snort) or human commentary.

## Data collection

The survey was created using Qualtrics (Qualtrics, Provo, UT, USA), a web-based survey platform. The survey was divided into four main sections: (1) Informed consent; (2) Participant demographics; (3) Categorisation of the 31 media pieces; and (4) the Multidimensional Assessment of Interoceptive Awareness (Version 2) (MAIA-2) (for survey questions, see S1 in the Supplementary material).

Participants were recruited using snowball sampling across relevant social media platforms (e.g. Facebook, Instagram and LinkedIn). Recruitment letters were distributed to university faculty and students via e-mail lists and advertisements were placed in equine newsletters (e.g. Equine Guelph). The survey was accessible either by a URL link or QR code. The survey was open to any participant aged 18 or older and previous experience with horses was not required. The survey was available from June 30 to July 27 2022 (total of 29 days).

After informed consent, the survey collected demographic data from participants including age, gender, education, country of residence and experience with horses. The survey then moved into the media categorisations, however prior to starting this section, participants were provided with the following definitions of positive, neutral and negative human-horse interactions:

**Positive interaction:** The horse exhibits a positive response to human interaction. The horse may seem attentive, engaged or appear to enjoy the interaction with the human.

**Neutral interaction:** The horse does not exhibit a noticeable response to human interaction. The horse may seem disengaged while still following human direction or participating in the interaction.

**Negative interaction:** The horse exhibits a negative response to human interaction. The horse may seem fearful, avoidant or attempt to stop the interaction.

The 31 pieces of media were presented to participants in randomised order. All nine images were uploaded directly onto Qualtrics and all 22 videos were uploaded to YouTube (www.youtube.com) then embedded in Qualtrics using HTML code. YouTube controls were left on, allowing the participants to replay the video as often as required before selecting their answer. Each piece of media was displayed independently from the others and once an answer had been submitted participants were unable to return to previous questions to change their answers. For each media piece, participants were asked to categorise the experience from the horse’s point of view as Positive, Likely Positive, Neutral, Likely Negative or Negative. To avoid biasing participant categorisation of media, the adjective ‘overt’ was omitted, and the adjective subtle was replaced with ‘likely’.

Two attention check questions were included within the media analysis section, roughly splitting it into three parts. The attention checks served to monitor the participant’s attention to detail and flag potential instances of the participants selecting answers at random before moving on to the next question.

Each media question collected data pertaining to the participant’s time budget while completing the survey which included the time of their first and last click per question, total click counts, and the time of page submission. At the end of the media section, an open-ended question asked participants to describe what behavioural clues from the horse they used to classify both positive and negative human-horse interactions (e.g. ear position).

The final part of the survey consisted of the Multidimensional Assessment of Interoceptive Awareness (Version 2) (MAIA-2) to assess participants’ interoception (Mehling *et al.* 2018). The MAIA-2 consists of 37 items and the participant selected how often they identified with each item on a scale of 0 to 5, 0 being never and 5 being always. For example, participants were presented with the following prompt: “When I feel pain or discomfort, I try to power through it”, then asked to rate the degree to which they relate to the prompt. This section was purposely placed last to avoid any ‘emotional warm-up’ the participants could have experienced, as participant self-reflection of their own feelings and emotions prior to media assessment could better prepare them to evaluate the horses’ reactions to human interaction. Of the 37 questions within the MAIA-2, each belonged to a subset of one to seven questions that paired with the one following eight factors: noticing, not-distracting, not-worrying, attention regulation, emotional awareness, self-regulation, body listening and trust. The MAIA-2 questions were presented in random order to each participant and can be seen in S2 in the Supplementary material.

### Data curation

The attention checks, in conjunction with the total time participants spent viewing the media, served as a means of response exclusion. If the participant spent less than the total video run time on the entire media section (approximately 3 min) it was assumed that the media were not viewed in their entirety and random answers were selected. In this case the participant’s data were removed. If the participant failed one or both attention checks, their data were flagged and compared to the total time spent in the section. The data from any participant that failed one or both attention checks and had a view time less than the media run time for that section were excluded. Some data-sets were incomplete, which represented participants who skipped questions and did not complete the entire survey prior to submission. If a participant’s data-set was only missing responses within the MAIA-2 section, their data were still used for analysis within the media section but excluded during subsequent analyses directly related to the MAIA-2. Incomplete questionnaires did not represent withdrawal of consent, as participants who wished to withdraw were instructed not to submit the survey and their data were not recorded. Withdrawal after submission was not possible as no identifying information was collected to link a response to a particular person.

### Data analysis

All analyses were conducted using SAS (v9.4, SAS Institute, Cary, NC, USA) with significance levels specified at α = 0.05. Expert categorisations of all media pieces were considered to be 100% correct. The ability of participants to accurately characterise the human-horse interactions compared to the expert categorisations was analysed using two criteria. The first criterion required the participant to exactly match the expert categorisation of any media piece (expert exact match). The second criterion accepted participant responses that were close to expert categorisations (expert close match): for example, if the experts categorised a media piece as a ‘positive’ interaction, participant responses of both ‘positive’ or ‘likely positive’ were accepted as correct. Similarly, if the experts categorised a media piece as ‘negative’, participant responses of both ‘negative’ or ‘likely negative’ were accepted as correct. If the experts categorised a media piece as ‘neutral’, participant responses of ‘neutral’, ‘likely negative’ or ‘likely positive’ were all accepted as correct. The percent agreement between the expert and participant categorisations for both criteria were separately analysed for each media piece using a Pearson’s Chi-squared test. A General Linear Mixed Model with participant as a random factor examined the main factors of participant age, gender, education and level of experience with horses on their percent agreement with expert categorisations under both criteria. Tukey-Kramer *post hoc* multiple comparisons further examined significant results between the percent agreement of participant responses relative to the experts and the main factors.

The MAIA-2 scores were calculated as described by Mehling *et al.* (2018) and as available in the public domain (www.osher.ucsf.edu/maia). Total scores represent the average of a six-point scale across all 37 questions and all scores ranged from zero to five. Each of the eight factors (e.g. Noticing) that contributed to the total MAIA-2 score also had a factor-specific score. Factor-specific scores were determined by taking the average response value across all factor-specific questions (e.g. Q1–Q4 are questions specific to the ‘Noticing’ factor). Higher total scores in the MAIA-2 represent a higher degree of interoception, and higher factor-specific scores represent a higher aptitude for each factor. Pearson’s product-moment correlations were run individually to determine any relationship with total and factor-specific MAIA-2 scores on the percent agreement between the participants’ and the experts’ categorisations of the media for both criteria.

Responses to the open-ended questions describing what behavioural clues from the horse participants used to classify both positive and negative human-horse interactions were analysed qualitatively using content analyses. Each response was coded using *a priori* themes of horse body parts (eyes, ears, face, head and neck, body, tail), engagement with or avoidance of the human, and general demeanour describing either positive or negative affective states. The frequency of the mention of each theme was calculated as the number of mentions divided by the total number of responses.

## Results

A total of 618 surveys were received, with 84 surveys excluded as being incomplete or failing the time or attention tests, resulting in 534 useable surveys. Not every participant answered every question as all questions were optional.

### Participant demographics

Participants (n = 534) were mostly females (n = 482; 90.3%) residing in Canada (n = 395; 75.2%). Participant age was fairly evenly distributed across categories (18–25 years: n = 111; 20.8%; 26–35 years: n = 122; 22.8%; 36–45 years: n = 84; 15.7%; 46–55 years: n = 77; 14.4%; > 55 years: n = 139; 26.0%). The majority of participants indicated they had advanced (n = 174; 32.6%) or expert (n = 199; 37.3%) level horse experience. Most participants were well-educated having completed a college or university degree (n = 196; 36.7%) or pursued further qualifications (n = 222; 41.6%), with 21.8% (n = 117) having no college or university education.

## Percent agreement of participants to experts’ categorisations

### Criterion 1

**Figure 1.**
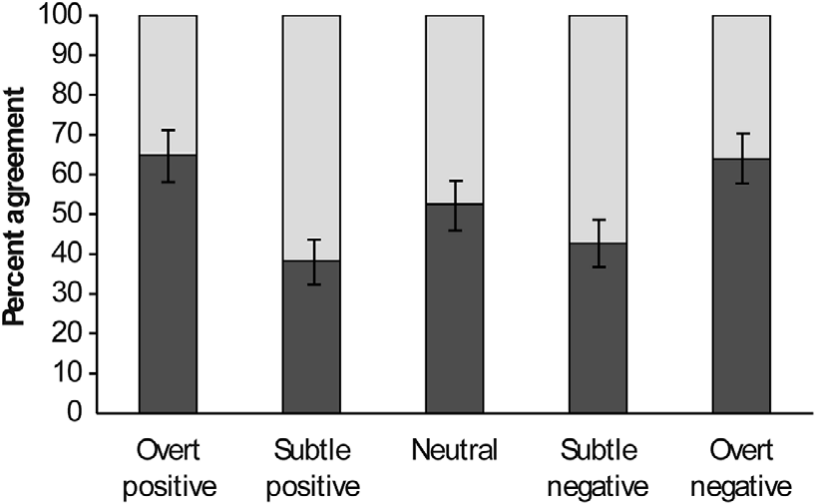
Mean percentage (dark grey bars [± SEM]) of human participants (n = 534) exactly matching categorisations by experts (considered to be 100% correct; light grey bars) of various positive, negative and neutral scenarios of human-horse interactions. Responses did not match expert categorisations in any of the categories (*P* < 0.0001).

Expert categorisations were considered 100% correct and under Criterion 1 participants had to match expert categorisations exactly. Participant categorisations ranged from 32.5–87.5% agreement and did not reflect expert exact matches for any of the 31 media pieces (χ^2^ = 1,392.8, df = 4; *P* < 0.0001). Overall expert exact matches by participants occurred 52.5% of the time. However, participants performed better for certain scenarios relative to others. On average, participants were better at identifying overt positive and overt negative human-horse interaction scenarios relative to subtle positive, neutral and subtle negative categorisations (Figure 1). Participant categorisations in only four questions exceeded 80% agreement to expert categorisations and these were all in the overt negative category (Q 27, 29, 30 and 31).

### Criterion 2

Expert categorisations were considered 100% correct and under Criterion 2 participants had to closely match expert categorisations. For example, if the participant selected likely positive but the correct answer was overt positive, it would still be considered correct. Participant percent agreement was higher under Criterion 2 compared to Criterion 1 (*P* < 0.0001 for all categories) but still did not closely match expert categorisation for any question (χ^2^ = 470.0, df = 3; *P* < 0.0001). On the whole, participants closely matched expert categorisations 78.5% of the time with a range of 15.3–98.5%. Participants were somewhat better at correctly identifying positive scenarios (80.4% agreement) compared to neutral (75.4% agreement) or negative (78.2% agreement) scenarios.

## Influence of participant age

### Criterion 1

Participants who were older than 55 years had lower percent agreement to expert exact matches of neutral scenarios (*F*_4,518_ = 5.24; *P* = 0.0004) compared to participants under 46 years. Participant age did not influence percent agreement in any other category (all *P* > 0.1000).

### Criterion 2

Participants who were older than 55 years had lower percent agreement to expert close matches of neutral (*F*_4,518_ = 3.73; *P* = 0.0053) and subtle negative (*F*_4,518_ = 3.74; *P* = 0.0052) scenarios compared to participants aged 26–35 years. Participant age did not influence percent agreement in any other category (all *P >* 0.2100).

## Influence of participant gender

### Criterion 1

Women outperformed men in exactly matching expert categorisations of overt positive (*F*_2,518_ = 3.83; *P* = 0.0223) human-horse interaction scenarios. Women tended to outperform men in exactly matching expert categorisations of subtle negative (*F*_2,518_ = 2.35; *P* = 0.0961) and overt negative (*F*_2,518_ = 3.35; *P* = 0.0963). Participant gender did not influence percent agreement in any other category (all *P* > 0.2200).

### Criterion 2

Women outperformed men in closely matching expert categorisations of overt positive (*F*_2,518_ = 3.75; *P* = 0.0242) and subtle negative (*F*_2,518_ = 5.92; *P* = 0.0029) human-horse interaction scenarios. Women tended to outperform men in closely matching expert categorisations of overt negative (*F*_2,518_ = 2.87; *P* = 0.0577) scenarios. Participant gender did not influence percent agreement in any other category (all *P* > 0.1200).

## Influence of participant level of education

### Criterion 1

**Figure 2.**
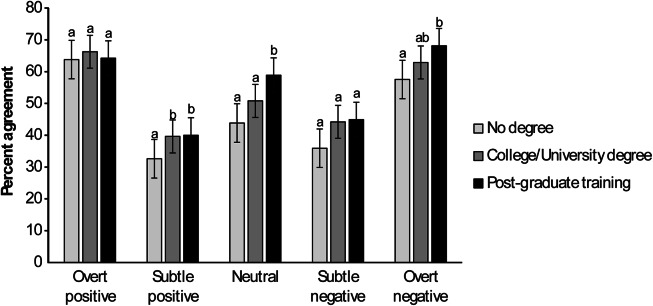
Mean (± SEM) percentages of human participants (n = 534) exactly matching expert categorisations (considered to be 100% correct) of various positive, negative and neutral scenarios of human-horse interactions according to their level of education. Within a category, bars with different superscripts (a,b) differ significantly (*P* < 0.031).

Education did not influence participants’ exact expert matches of overt positive (*F*_2,518_ = 0.73; *P* = 0.4801) and subtle negative (*F*_2,518_ = 1.04; *P* = 0.3542) human-horse interaction scenarios compared to expert categorisations. However, participants with more education did better at exactly matching expert categorisations of subtle positive (*F*_2,518_ = 3.52; *P* = 0.0304), neutral (*F*_2,518_ = 11.60; *P* < 0.0001) and overt negative (*F*_2,518_ = 4.52; *P* = 0.0114) scenarios than those with less education (Figure 2).

### Criterion 2

Participants with more education did better at closely matching expert categorisations of neutral (*F*_2,518_ = 13.18; *P* < 0.0001), subtle negative (*F*_2,518_ = 4.34; *P* = 0.0135) and overt negative (*F*_2,518_ = 3.73; *P* = 0.0246) scenarios than those with less education. Participant education did not influence percent agreement in any other category (all *P* > 0.1000).

## Influence of horse experience

### Criterion 1

**Figure 3.**
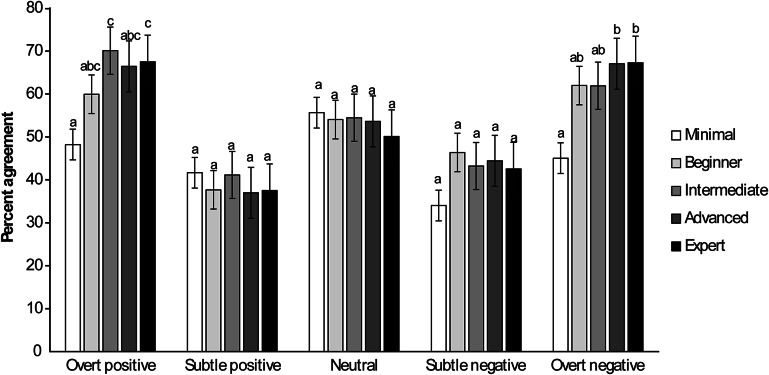
Mean (± SEM) percentages of human participants (n = 534) exactly matching categorisations by experts (considered to be 100% correct) of varying depictions of positive, negative and neutral human-horse interactions relative to their level of horse experience. Within a category, bars with different superscripts (a,b,c) differ significantly (*P* < 0.03).

Participants with more experience with horses performed better at exactly matching the experts than those with less horse experience when categorising both overt positive (*F*_4,518_ = 2.72; *P* = 0.0290) and overt negative (*F*_4,518_ = 4.38; *P* = 0.0017) human-horse interaction scenarios (Figure 3). Horse experience did not affect the percentage of exact matches of subtle positive, neutral or subtle negative scenarios (all *P* > 0.2770).

### Criterion 2

Participants with more experience with horses performed better at closely matching the experts than those with less horse experience when categorising subtle negative (*F*_4,518_ = 6.87; *P* < 0.0001) human-horse interaction scenarios. Participants with more experience with horses tended to perform better at closely matching the experts than those with less horse experience when categorising subtle positive (*F*_4,518_ = 2.19; *P* = 0.0689) human-horse interaction scenarios. Horse experience did not affect the percentage of closely matched categorisations of overt positive, neutral or overt negative scenarios (all *P* > 0.5735).

## Influence of MAIA-2 scores

Participant responses to the MAIA-2 are found in Table 2. Participant scores for any of the MAIA-2 factor-scales were not correlated to the percent agreement of categorisations of various scenarios of human-horse interactions compared to expert categorisations for either exact matches (Criterion 1; *r* = 0.087, n = 455; *P* = 0.0631) or close matches (Criterion 2; *r* = –0.062, n = 455; *P* = 0.1989).

**Table 2.** Average human participant (n = 534) scores for each factor scale answered within the Multidimensional Assessment of Interoceptive Awareness questionnaire (MAIA-2). Scores ranged from 0–5 with higher scores indicating higher interoception for that scale

MAIA factor scale	Mean	SD
Total score	4.08	0.617
Noticing	4.64	0.888
Not distracting	3.21	1.044
Not worrying	3.78	0.947
Attention regulation	4.13	0.811
Emotional awareness	4.36	0.816
Self regulation	3.76	1.027
Body listening	4.16	1.008
Trusting	4.56	0.864

## Qualitative analysis

Participants were asked to describe what clues from the horse’s behaviour they used to categorise both positive and negative human-horse interactions. Responses involving the ears, eyes, face, head and neck, body, tail and movement appeared in both the positive and negative descriptors provided by participants (Table 3).

**Table 3.** Responses provided by survey participants (n = 457) for both positive and negative indicators of horse affective state after viewing media pieces (n = 31). Frequencies represent the number of participants (n; %) who indicated that they utilised these physical horse traits to categorise human-horse interactions during the media analysis. Qualifiers are examples of the qualitative descriptions respondents wrote

Comment	Positive frequency	Positive qualifiers	Negative frequency	Negative qualifiers
**Ears**	n = 308; 67.0%	Forward, relaxed, soft, toward person, up, floppy, straight,	n = 272; 60.0%	Back, pinned, fixed, pressed
**Eyes**	n = 147; 32.2%	Soft, relaxed, calm, curious, on human, closing, interested, gentle, kind, quiet, sleepy	n = 191; 41.9%	Whale eye, open wide, bulging, alert, empty, tension, hard, rolling, anxious, no eye contact
**Face**	n = 131; 28.7%	Lick and chew, relaxed facial muscles, loose jaw/lips, facial expression, soft	n = 90; 19.7%	Nostrils flared, curling lips, mouth clamped, tight lips, facial tension, pain face, grimace
**Head & neck**	n = 110; 24.1%	Neutral position, head low, relaxed, neck stretched toward person	n = 143; 31.4%	Head tossing, raised head, head shy, tension in neck, fussy with head
**Body**	n = 112; 24.5%	Relaxed posture, engaged, inviting, no visible tension, soft, body language	n = 93; 20.4%	Tense posture, stiff, increased muscle tone, body language, body leaning away
**Tail**	n = 14; 3.1%	Still, quiet, swaying, wagging, relaxed	n = 39; 9.0%	Flicking, raised, tense, swishing, whipping, clamped
**Movement**	n = 42; 9.2%	Standing still, movement toward person, standing near person, moves calmly, relaxed movement	n = 261; 57.2%	Escape movements, frozen, jerky, agitated, flight response, skittish, frantic, sudden, pulling away, quick, jumpy

Along with body parts, participants also referred to whether the horse was engaged with the human (n = 271; 59.3%) during positive interactions or avoided (n = 229; 50.2%) the human during negative interactions. Engagement was described as seeking human attention, voluntarily approaching, leaning in or staying close to the human, initiating contact, nuzzling, doing what the human asked of them and their ability to leave if they chose. Seventeen respondents (3.7%) specifically referred to the horse as ‘happy’ while the majority of respondents (n = 286; 62.6%) described the demeanour of the horse in other ways, including curious, inquisitive, calm and relaxed. A small number of respondents (n = 11; 2.4%) explicitly mentioned the actions of the human contributing to their assessment of the scenario, such as maintaining a loose lead rope connection, being relaxed themselves or providing treats or release cues to the horse.

Avoidance was described as being evasive, looking away, pulling back, trying to get away from human, displaying defensive reactions or being uncooperative to the human’s request. The demeanour of the horse included descriptors such as fear, tension, disinterest, jaded, aggression, stress, anxious, inhibited, reactive, unsure, unhappy, irritated, apprehensive and distant. Fifteen respondents (3.3%) indicated the actions of the human being responsible for the horse’s response, either by the human pulling on the reins or using a lot of tension on the lead rope, forcing the horse to do something they did not want to do or using tools to restrict motion, not ensuring the horse understood their request, not being aware of the horse’s reactions or being rushed.

## Discussion

The results presented here corroborate findings from other researchers in that generally humans are not very successful in distinguishing the affective state of horses during their interactions with humans (Bell *et al.* 2019; Rogers & Bell 2022; Luke *et al.* 2023). Participants did better at correctly characterising the valence of the interactions as positive or negative but may have lacked the finesse required to observe the more subtle signs. In particular, neutral responses to human interaction were challenging for the participants to identify in horses. Age, gender, education and horse experience all affected the participants’ ability to categorise the horses’ affective states. Older participants struggled more to correctly categorise neutral scenarios, women generally outperformed men, and those with more education and more horse experience were better able to categorise certain scenarios. How well a participant was in touch with their own inner bodily sensations did not impact their categorisations of the affective states of the horses. Participants provided many descriptors of horse behaviour to qualify their categorisations, mostly relying upon the ears and eyes of the horse and how engaged they were with the human. While participants were able to define various behaviours the horse might portray, there seemed to be a disconnect between recognising the behaviours and linking them to affective states.

In this study, the expert categorisations were taken as 100% correct, although others have shown that experts do not always agree. Pearson *et al.* (2021) showed a poor agreement among equine behaviour experts and veterinarians when assessing behavioural indicators of stress in horses undergoing veterinary treatment. Bell *et al.* (2019) showed imperfect consensus of horse affective state among six equine behaviour experts rating videos. However, Pannewitz and Loftus (2023) reached consensus among 30 equine behaviour experts on behavioural indicators of frustration in horses. Young *et al.* (2012) found the ratings of thirteen equine professionals on the behavioural responses of horses in various stressful situations (e.g. clipping, fireworks) using a simple behaviour scale ranging from no stress (1) to extreme stress (10) to reliably correspond to both heart rate and cortisol measures from those horses. Since at least six of our seven (> 85%) experts had to agree on the behavioural categorisation of the media, and those media that did not reach that benchmark were not included in the survey, we can be fairly confident that the included media depicted the assigned affective state. However, this resulted in our media not being divided evenly among the various categories, with fewer media pieces in slightly negative scenarios. This could imply that even our experts had difficulty interpreting these scenarios since there were fewer media pieces agreed upon in this category.

On average, our survey participants matched expert categorisations of the affective states of the horses depicted in the media only 52.5% of the time, which does not differ much from chance. Similar to Bell *et al.* (2019), participants were better at recognising overt positive and negative behaviours the horses displayed but were less discerning of the subtle behaviours. This lack of awareness of subtle signals can lead to escalation of dangerous behaviours and compromise both human and horse safety (Bell *et al.* 2019; Rogers & Bell 2022).

When analysed under broader standards (i.e. Criterion 2), our survey participants’ responses improved although they still did not match the expert responses. Under this less stringent analysis, it was clear the participants recognised the valence of the horses’ affective states as positive or negative even if they missed the more subtle signals. Participants struggled most with identifying neutral situations where the horse did not appear responsive to or engaged with the human, highlighting how difficult it can be to accurately identify both subtle and neutral horse behaviours. A horse’s response to human interaction is a combination of their own temperament, previous experiences with humans, individual coping styles and training (Wechsler 1995; Seaman *et al.* 2002; McGreevy *et al.* 2009; Bell *et al.* 2019; Hausberger *et al.* 2021). These factors may lead to minimal behavioural responses that may mask the horse’s affective state. It could be that participants misidentified neutral human-horse interactions to be more positively valanced (Lesimple & Hausberger 2014; Bell *et al.* 2019). If the horse depicted in the neutral media was a passive coper, this may improperly translate to humans as the horse being calm or relaxed, which are desirable traits (Bell *et al.* 2019). Recent research suggests that relying solely upon physical behaviours may not be a reliable indicator of horse affect during stressful situations or interactions as physiological measures may still indicate a stress response (Squibb *et al.* 2018). For the purposes of this study, human-horse interactions depicting a horse that was disengaged from or minimally participating in the interaction with a human was assigned a neutral categorisation by the experts, but this highlights that despite expert consensus, it may still be an inaccurate categorisation of the true valance of affect being experienced by the horse.

In general, age did not influence the participants’ ability to match expert categorisation of human-horse interactions with the exception of those older than 55 years performing poorer than younger participants when categorising neutral (Criteria 1 and 2) and subtle negative (Criterion 2) scenarios. Older participants viewing photographs of cats were similarly less successful in identifying positive or negative affect than younger participants (Dawson *et al.* 2019). Others have shown that the ability to recognise emotions in human facial expressions decreased with age, most particularly when regarding images of neutral or negative emotions (Malykhin *et al.* 2023).

In this study, women outperformed men in correctly categorising obvious positive and negative affective states in horses and tended to outperform men in correctly categorising subtle indicators of affective state. The participants in this study represented more females than males, which is typical of the equine industry in general (Dashper 2016; Fenner *et al.* 2019). More women than men believe that horses can feel emotions (Hötzel *et al.* 2019) and this, together with the fact that women are more empathic toward animals than men (e.g. Christov-Moore *et al.* 2014), may account for this result. Similarly, women were better at decoding the valence of horse whinnies than men (Merkies *et al.* 2021).

Survey participants with a higher level of education were better able to categorise some horse affective states than those with less education. However, Pannewitz and Loftus (2023) showed no difference among equine behaviour professionals with or without a PhD in identifying behavioural indicators of frustration in horses. Similarly, education was not found to be a significant factor in human ability to distinguish affective states in cats (Dawson *et al.* 2019). Formal education in general is believed to increase human cognitive abilities and was shown to be a stronger factor than experience in recognition of the importance of ecosystems (Lima & Bastos 2019). However, the influence of formal education on recognition of horse behaviours remains inconclusive.

Within the overt positive and negative categories, participants with more horse experience did perform better than those with little horse experience, although this pattern did not appear in the subtle positive, neutral or subtle negative categories. Horse experience has been related to the ability to distinguish horse behaviours with conflicting outcomes. Fox (2023) showed that lay people performed just as well as practitioners of equine-assisted services when describing the affective states of horses in videos even though they reported a lower level of horse experience. Contrarily, Braun *et al.* (2024) showed that those with more horse experience did better at characterising the affective state of horses in photographs compared to inexperienced people, although they still only correctly identified affective state 50% of the time. Fourth year veterinary students were better at characterising horse behaviour than first year students with less self-reported horse experience (Guinnefollau *et al.* 2019). Participants with more experience performed better when assessing the videos depicting in-hand dressage and behavioural rehabilitation but not when assessing ridden horse videos (Bell *et al.* 2019). It could be that experience interplays with cognitive bias – we process information and make decisions based upon our own perceptions and memories, which are led by our experiences (Azzopardi 2021). Those who spend extended periods of time with horses tend to overconfidently assess their horses’ affective state as positive and misinterpret indicators of negative affect (Lesimple & Hausberger 2014; Bell *et al.* 2019; Bornmann *et al.* 2021) and those involved in the daily care of horses underestimate the expression of negative affect as they are likely desensitised to behaviours that, in their opinion, do not affect a horse’s overall well-being (Lesimple & Hausberger 2014).

Empathy is the ability to recognise the affective state of another and to respond with appropriate emotion. Despite there being a close relationship between empathy seen between humans and interoception (Ernst *et al.* 2013; Hübner *et al.* 2021) results from this study showed no correlation between participant interoception as assessed by the MAIA-2 and their ability to correctly categorise the affective states of horses interacting with humans. The MAIA-2 is a commonly used measure of interoception (Desmedt *et al.* 2022; Vig *et al.* 2022) and has demonstrated relationships to interpreting facial expressions of others (Hübner *et al.* 2021). To the authors’ knowledge, the MAIA-2 has not been used to evaluate interpretation of affective states of animals. The lack of correlation between participants’ interoception and their ability to correctly categorise human-horse interactions could indicate that the MAIA-2 is not transferrable across species, as even experts struggle to reach a consensus regarding which behavioural markers suggest a horse may be stressed (Pearson *et al.* 2021). An alternate explanation is that people’s perceptions of their own thoughts and emotions are more subjective than their perception of others, as observed within the human medical field where practitioners tend to underestimate their patients’ pain (Marquié *et al.* 2003).

Typically, those actively involved in equestrian activities pride themselves on being able to read their horse, often attributing anthropomorphic states to their horse’s expression of emotion (Hötzel *et al.* 2019). Our results did not show much evidence of anthropomorphism in the qualitative responses provided by participants. Despite performing poorly in recognising subtle positive and negative categories, some survey participants were still able to describe subtle indicators of affect (e.g. muscular tension). However, most qualitative descriptions were either excessively broad (e.g. ears, eyes) or referred to more overt indicators of behaviour (e.g. biting). Similarly, Bell *et al.* (2019) concluded that participants were more likely to list overt indicators of behaviour although some would be able to identify more subtle cues, like changes in eye aperture (Bell *et al.* 2019). Survey participants gauged horse affective state mainly in terms of expression of specific body parts. In particular, the eyes and ears were referred to most often as indicative of how a horse was feeling. More body descriptors were suggested to evaluate negative affective state (e.g. head and neck position) than positive affective state, and more comments in general were provided for negative states. This supports the idea that identifying positive affective states in horses is much more challenging than identifying negative affective states (Zeitler-Feicht *et al.* 2024). Participants also voiced more concern over human actions creating or exacerbating the scenarios leading to negative affective states in the horse than scenarios leading to positive affective states. It is reassuring that survey participants were able to recognise the harmful actions of humans in the scenarios but disappointing that overall they were still unable to characterise when horses were experiencing negative affective states. A similar study evaluating human ability to recognise distress in horses also found that some participants would be comfortable with their horse being subjected to the same treatment or situation that could result in negative affect (Bell *et al.* 2019).

Rapid advances in technology make it now increasingly possible to decipher horse behaviour using artificial intelligence. Simple detection of key body points and their change over time allows for gross determination of animal activity as an indicator of health and well-being (Kleanthous *et al.* 2022). More sophisticated approaches utilise deep learning to determine patterns and features specific to affective states reflecting potential pain in horses based on more frequent changes in posture (Martin-Cirera *et al.* 2024) and the Horse Grimace Scale (Lencioni *et al.* 2021). While these techniques are very promising for behaviour recognition and would drastically reduce time commitment for assessment in a non-invasive manner, the methodology still remains in its infancy (Rohan *et al.* 2024).

## Animal welfare implications

To safeguard both human and horse welfare during human-horse interactions, it is imperative that humans recognise positive human-horse interactions to promote their reoccurrence, and negative human-horse interactions to avoid subjecting horses to unnecessary stress. However, similar to other studies, our survey participants were unable to successfully deduce horse affective state in various scenarios compared to expert categorisations. Participants were more successful at identifying overt indicators of affect over subtle indicators and could generally infer the positive or negative valence of a scenario. Human literature suggests that those who are more aware of their own internal body sensations may have more empathy for others. To our knowledge, this is the first study that has attempted to relate human interoception to identification of horse affective states. However, our results showed participants’ interoception determined by the MAIA-2 questionnaire did not impact their ability to match expert categorisations of human-horse interactions. Despite participants being able to qualitatively describe negative affect in horses, this did not translate to their ability to characterise affective state, underscoring the subconscious disconnect between behavioural signals and affective states. Future research would benefit from focusing on subtle indicators of horse affect, to identify if participants misconstrue subtle indicators of negative affect as neutral, or even positive, as many involved in horse care inaccurately identify or ignore negative indicators of affect. Although participants with more self-reported experience with horses performed better at identify affective states, continued improvement in education and awareness of horse behaviour will ensure continued improvement for horse welfare.

## Supporting information

Merkies and Trudel supplementary material Merkies and Trudel supplementary material

## Supplementary material

The supplementary material for this article can be found at http://doi.org/10.1017/awf.2024.55.
