# Supplementary material for: How well can you tell? Success of human categorisation of horse behavioural responses depicted in media
Source: Anim Welf. 2024 Nov 11;33:e50. doi: 10.1017/awf.2024.55 (PMC11589072; doi:10.1017/awf.2024.55)
Supplement: Merkies and Trudel supplementary material [file S0962728624000551sup001.pdf]

How well can you tell? Success of human categorisation of horse behavioural responses depicted in media

Katrina Merkieshttps://orcid.org/0000-0002-9462-4150[a,b], Katelyn Trudel[a,b]

[a] Department of Animal Biosciences, University of Guelph, Guelph ON N1G 2W1, Canada

[b] Campbell Centre for the Study of Animal Welfare, University of Guelph Guelph, ON N1G 2W1, Canada

Author for correspondence: Katrina Merkies, email: kmerkies@uoguelph.ca

S1

Human Categorisation of Horse-Human Interactions (REB 21-12-026)

Q 1 How old are you?

- ◯ 18-25 years old
- ◯ 26-35 years old
- ◯ 36-45 years old
- ◯ 46-55 years old
- ◯ 56-65 years old
- ◯ Greater than 65 years old

Q 2 What is your gender?

- ◯ Woman
- ◯ Man
- ◯ My gender identity is not listed above (please specify) ____________
- ◯ Choose not to respond

Q 3 What is your level of experience with horses?

◯ No experience with horses

◯ Minimal (e.g. I have ridden or been around horses once or twice)

◯ Beginner (e.g. I have taken a few riding lessons and I've been around horses on multiple occasions)

◯ Intermediate (e.g. I ride horses often and I am around them frequently)

◯ Advanced (e.g. I have been riding horses for a long time and I am around them multiple times a week)

◯ Expert (e.g. I have been riding horses for most of my life and I am around them almost every day)

Q 4 What is your highest level of education?

◯ Some high school

◯ Completed high school

◯ Some college/university

◯ Apprenticeship training and trades

◯ Completed college/university

◯ Some graduate education

◯ Completed graduate education

◯ Professional degrees

Q 5 In which country do you currently reside?

▼ Afghanistan (1) ... Zimbabwe (1357)

In this section, you will be presented with either a video or an image of a horse-human interaction. The videos can be viewed multiple times and contain no audio.

Using the following definitions of positive, neutral, and negative interactions, please categorise each interaction from the ***horse's point of view***:

Positive Interaction: The horse exhibits a positive response to human interaction. The horse may seem attentive, engaged, or appear to enjoy the interaction with the human.

Neutral Interaction: The horse does not exhibit a noticeable response to human interaction. The horse may seem disengaged, while still following human direction or participating in the interaction.

Negative Interaction: The horse exhibits a negative response to human interaction. The horse may seem fearful, avoidant, or attempt to stop the interaction.

Please answer the following questions using the options provided. Once you have moved on to the next question, you cannot return to previous questions to modify your responses.

Q 6-37 What kind of interaction is this?

- ○ Positive
- ○ Likely Positive
- ○ Neutral
- ○ Likely Negative
- ○ Negative

Attention Check #1 (appearing after Media Q10)

How many times has a cow appeared in the included media?
This is to check your attention, please select 7 as the answer.

- ○ 1
- ○ 7
- ○ 8
- ○ 3
- ○ 6

Attention Check #2 (appearing after Media Q20)

Which four-legged animal is being shown repeatedly in this section?
This is to check your attention, please select Chicken as the answer.

- ○ Sheep
- ○ Cat
- ○ Cow
- ○ Horse

○ Chicken

Descriptor Words (Positive & Negative)

Q 38 What clues from the horse's behaviour did you use to classify ***POSITIVE*** horse-human interactions? (written text)

Q 39 What clues from the horse's behaviour did you use to classify ***NEGATIVE*** horse-human interactions? (written text)

Multidimensional Assessment of Interoceptive Awareness MAIA :

In this section, you will find a list of statements. Please indicate how often each statement applies to you generally in daily life.

These results will be indicative your personal qualities, like emotional awareness or self-regulation, and linked to your categorisation of the horse-human interactions shown in this survey. We are interested to know how awareness of your own body and feelings may influence your sensitivity to how animals are feeling. Read through each of the 37 following statements and indicate how often you feel this way in your daily life.

	Never (0)	(1)	(2)	(3)	(4)	Always (5)
When I am tense I notice where the tension is located in my body.	○	○	○	○	○	○
I notice when I am uncomfortable in my body.	○	○	○	○	○	○
I notice where in my body I am comfortable.	○	○	○	○	○	○
I notice changes in my breathing, such as whether it slows down or speeds up.	○	○	○	○	○	○
I ignore physical tension or discomfort until they become more severe.	○	○	○	○	○	○
I distract myself from sensations of discomfort.	○	○	○	○	○	○
When I feel pain or discomfort, I try to power through it.	○	○	○	○	○	○
I try to ignore pain	○	○	○	○	○	○

I push feelings of discomfort away by focusing on something.	○	○	○	○	○	○
When I feel unpleasant body sensations, I occupy myself with something else so I don't have to feel them.	○	○	○	○	○	○
When I feel physical pain, I become upset.	○	○	○	○	○	○
I start to worry that something is wrong if I feel any discomfort.	○	○	○	○	○	○
I can notice an unpleasant body sensation without worrying about it.	○	○	○	○	○	○
I can stay calm and not worry when I have feelings of discomfort or pain.	○	○	○	○	○	○
When I am in discomfort or pain I can't get it out of my mind.	○	○	○	○	○	○
I can pay attention to my breath without being distracted by things happening around me.	○	○	○	○	○	○
I can maintain awareness of my inner bodily sensations even when there is a lot going on around me.	○	○	○	○	○	○
When I am in conversation with someone, I can pay attention to my posture.	○	○	○	○	○	○
I can return awareness to my body if I am distracted.	○	○	○	○	○	○
I can refocus my attention from thinking to sensing my body.	○	○	○	○	○	○
I can maintain awareness of my whole body even when a part of me is in pain or discomfort.	○	○	○	○	○	○

I am able to consciously focus on my body as a whole.	○	○	○	○	○	○
I notice how my body changes when I am angry.	○	○	○	○	○	○
When something is wrong in my life I can feel it in my body.	○	○	○	○	○	○
I notice that my body feels different after a peaceful experience.	○	○	○	○	○	○
I notice that my breathing becomes free and easy when I feel comfortable.	○	○	○	○	○	○
I notice how my body changes when I feel happy / joyful.	○	○	○	○	○	○
When I feel overwhelmed I can find a calm place inside.	○	○	○	○	○	○
When I bring awareness to my body I feel a sense of calm.	○	○	○	○	○	○
I can use my breath to reduce tension.	○	○	○	○	○	○
When I am caught up in thoughts, I can calm my mind by focusing on my body/breathing.	○	○	○	○	○	○
I listen for information from my body about my emotional state.	○	○	○	○	○	○
When I am upset, I take time to explore how my body feels.	○	○	○	○	○	○
I listen to my body to inform me about what to do.	○	○	○	○	○	○
I am at home in my body.	○	○	○	○	○	○
I feel my body is a safe place.	○	○	○	○	○	○
I trust my body sensations.	○	○	○	○	○	○

S2

Multidimensional Assessment of Interoceptive Awareness (Version 2) (MAIA-2): Questionnaire and Scoring Instructions. Note: Reverse scoring (R) (5 – x) items 5, 6, 7, 8, 9 and 10 on Not-Distracting, and items 11, 12 and 15 on Not- Worrying. (Mehling *et al.* 2018)

**Question**	**Prompt**	**Scoring**					
		**Never**					**Always**
Q1	When I am tense I notice where the tension is located in my body.	0	1	2	3	4	5
Q2	I notice when I am uncomfortable in my body.	0	1	2	3	4	5
Q3	I notice where in my body I am comfortable.	0	1	2	3	4	5
Q4	I notice changes in my breathing, such as whether it slows down or speeds up.	0	1	2	3	4	5
Q5	I ignore physical tension or discomfort until they become more severe.	0	1	2	3	4	5
Q6	I distract myself from sensations of discomfort.	0	1	2	3	4	5
Q7	When I feel pain or discomfort, I try to power through it.	0	1	2	3	4	5

Q8	I try to ignore pain	0	1	2	3	4	5
Q9	I push feelings of discomfort away by focusing on something	0	1	2	3	4	5
Q10	When I feel unpleasant body sensations, I occupy myself with something else so I don't have to feel them.	0	1	2	3	4	5
Q11	When I feel physical pain, I become upset.	0	1	2	3	4	5
Q12	I start to worry that something is wrong if I feel any discomfort.	0	1	2	3	4	5
Q13	I can notice an unpleasant body sensation without worrying about it.	0	1	2	3	4	5
Q14	I can stay calm and not worry when I have feelings of discomfort or pain.	0	1	2	3	4	5
Q15	When I am in discomfort or pain I can't get it out of my mind	0	1	2	3	4	5
Q16	I can pay attention to my breath without being distracted by things happening around me.	0	1	2	3	4	5
Q17	I can maintain awareness of my inner bodily sensations even when there is a lot going on around me	0	1	2	3	4	5
Q18	When I am in conversation with someone, I can pay attention to my posture.	0	1	2	3	4	5
Q19	I can return awareness to my body if I am distracted.	0	1	2	3	4	5
Q20	I can refocus my attention from thinking to sensing my body.	0	1	2	3	4	5
Q21	I am able to consciously focus on my body as a whole.	0	1	2	3	4	5
Q22	I notice how my body changes when I am angry.	0	1	2	3	4	5
Q23	When something is wrong in my life I can feel it in my body.	0	1	2	3	4	5
Q24	I notice that my breathing becomes free and easy when I feel comfortable.	0	1	2	3	4	5
Q25	I notice how my body changes when I feel happy / joyful.	0	1	2	3	4	5
Q26	When I feel overwhelmed I can find a calm place inside.	0	1	2	3	4	5
Q27	When I bring awareness to my body I feel a sense of calm.	0	1	2	3	4	5
Q28	I can use my breath to reduce tension.	0	1	2	3	4	5
Q29	I listen for information from my body about my emotional state.	0	1	2	3	4	5

Q30	When I am upset, I take time to explore how my body feels.	0	1	2	3	4	5
Q31	I listen to my body to inform me about what to do.	0	1	2	3	4	5
Q32	I am at home in my body.	0	1	2	3	4	5
Q33	I feel my body is a safe place.	0	1	2	3	4	5
Q34	I can maintain awareness of my whole body even when a part of me is in pain or discomfort.	0	1	2	3	4	5
Q35	I notice that my body feels different after a peaceful experience.	0	1	2	3	4	5
Q36	When I am caught up in thoughts, I can calm my mind by focusing on my body/breathing.	0	1	2	3	4	5
Q37	I trust my body sensations.	0	1	2	3	4	5

**Factor**	**Scoring Equation**
Noticing	(Q1 + Q2 + Q3 + Q4) / 4
Not Distracting	(Q5(R) + Q6(R) + Q7(R) + Q8(R) + Q9(R) + Q10(R)) / 6
Not Worrying	(Q11(R) + Q12(R) + Q13 + Q14 + Q15(R)) / 5
Attention Regulation	(Q16 + Q17 + Q18 + Q19 +Q20 + Q21 + Q22) / 7
Emotional Awareness	(Q23 + Q24 + Q25 + Q26 + Q27) / 5
Self-regulation	(Q28 + Q29 + Q30 + Q31) / 4
Body listening	(Q32 + Q33 + Q34) / 3
Trusting	(Q35 + Q36 + Q37) / 3
